# Factors contributing to musculoskeletal disorders and neck–trunk posture among Shiraz university of medical sciences students using Digimizer

**DOI:** 10.1038/s41598-026-53292-y

**Published:** 2026-05-21

**Authors:** Zahra Zamanian, Sanaz Karimpour, Hadi Daneshmandi, Sanaz Amiri, Arman Amiri

**Affiliations:** 1 Department of Occupational Health and Safety Engineering, School of Health, Shiraz University of Medical Sciences, Shiraz, Iran; 2 Department of Occupational Health and Safety Engineering, School of Health, Abadan University of Medical Sciences, Abadan, Iran; 3 Research Center for Health Sciences, Institute of Health, Shiraz University of Medical Sciences, Shiraz, Iran; 4 Student Research Committee, Shiraz University of Medical Sciences, Shiraz, Iran

**Keywords:** Posture, Ergonomics, Lighting, Neck, Trunk, Students, Health care, Health occupations, Risk factors

## Abstract

Musculoskeletal disorders are common among students exposed to prolonged sitting, and objective posture assessment is essential to clarify the contribution of ergonomic and environmental factors. This study aimed to evaluate factors associated with neck and trunk posture using quantitative image-based analysis. In this descriptive-analytical cross-sectional study, 25 students from Shiraz University of Medical Sciences were assessed. Neck and trunk angles were extracted from standardized lateral images using Digimizer software. Ergonomic compatibility between chairs and students’ anthropometric characteristics was evaluated through a 12-item checklist. Musculoskeletal discomfort was assessed using the Cornell questionnaire, and mental workload was measured by NASA-TLX. General and task lighting indices and luminance were recorded. Correlation analyses and multiple linear regression were performed to determine predictors of posture angles. No significant associations were observed between demographic variables, lighting indices, mental workload, and neck or trunk angles (*p* > 0.05). In contrast, inappropriate seat height was associated with increased neck angle (*p* = 0.046), while insufficient seat width and lack of lower limb support were significantly related to increased trunk angle (*p* = 0.009 and *p* = 0.020). Trunk flexion was also associated with greater trunk angle (*p* = 0.035). Discomfort was most prominent in the neck and back regions. These findings indicate that postural deviations during study are primarily driven by chair design and anthropometric mismatch rather than lighting conditions or cognitive load.

## Introduction

Musculoskeletal disorders (MSDs) are health conditions that cause pain or injury in the muscles, bones, joints, tendons, ligaments, nerves, or supporting structures of the body, and they are among the leading global causes of disability, affecting over 1.7 billion people worldwide^1^. Such musculoskeletal injuries constitute a substantial proportion of work-related physical complaints across industrial settings as well as administrative, educational, and service-related activities. Consequently, MSDs, through their adverse effects on workforce health and the generation of direct and indirect costs for organizations, can exert a considerable economic burden^2^. The occurrence of MSDs in educational environments has increased in recent years, particularly among student populations who are exposed to prolonged static postures during study activities.^3^ Repetitive activities, inappropriate working posture, contact stress, excessive force, vibration, physical fatigue, and unfavorable workstation conditions are among the recognized factors contributing to the development of MSDs^4^. These factors often interact with one another, suggesting that musculoskeletal disorders should be examined within a multifactorial framework rather than being attributed to a single cause. Accordingly, evidence indicates that MSDs are multifactorial in nature, and various physical, ergonomic, and environmental conditions may contribute to their development. Among the various physical, ergonomic, and environmental factors influencing posture and musculoskeletal health, conditions related to the visual environment have also received attention in previous studies^5^. Dissatisfaction among individuals in office and educational settings is associated with poor or inadequate lighting, which is one of several physical environmental factors in the work environment^6^. Adequate lighting, along with other ergonomic and environmental conditions, is considered a health and safety factor contributing to optimal working conditions in workplaces^7^. Various characteristics of the physical environment, including illuminance level, light wavelength, lighting distribution, and luminance on visible surfaces, can influence visual comfort during task performance^8^. On the other hand, improper lighting system design may contribute to adverse outcomes such as fatigue, headaches, visual disturbances, impaired object and color discrimination, and an increased risk of accidents, while excessive light and glare may also result in ocular damage^9^. In addition to its negative visual and non-visual effects, inadequate lighting may reduce productivity, increase work-related absenteeism, contribute to accidents, and potentially promote the development of musculoskeletal disorders, particularly in combination with other ergonomic risk factors^10–13^. Accordingly, findings from several studies have demonstrated that visual fatigue is associated with discomfort in the upper body, and in environments with poor lighting conditions, visual system adaptation during tasks requiring high precision increases muscular tension in the upper extremities^14^. Given that adults rely on visual input for a substantial portion of their daily activities, sustained visual demands may influence postural behavior during work and study tasks^15^. The risk of upper extremity disorders may increase, and body posture may change due to inadequate lighting, especially when sustained for prolonged periods^16^. When individuals are exposed to an external stressor, they tend to modify their body posture to reduce this stress. Lighting is one of the physical factors that may influence body posture by prompting individuals to adopt compensatory head, neck, and trunk positions in order to achieve visual comfort during task performance^17^. However, the development of postural deviations and musculoskeletal disorders is rarely influenced by a single environmental factor and is more commonly the result of combined ergonomic, individual, and environmental exposures. Body posture, particularly sustained neck and trunk positions during prolonged study activities, represents a key biomechanical pathway through which multiple risk factors may contribute to musculoskeletal disorders. Therefore, accurate assessment of posture is essential for understanding the mechanisms underlying MSDs development in student populations.

In the assessment of individual posture, various approaches are employed, including self-reported, observational, and instrument-based methods. Self-reported and paper-based methods are subject to limitations such as observer bias and low inter- and intra-rater reliability. These limitations are substantially reduced in direct and instrument-based approaches, which provide higher accuracy and validity^18^. In this context, the use of software such as Digimizer, which enables precise and quantitative analysis of static images obtained from individuals’ body posture, has been introduced as an efficient and reliable tool for posture assessment^19^. This software allows accurate measurement of body angles and provides robust quantitative data for analyzing posture status and musculoskeletal problems. Moreover, the use of Digimizer minimizes human error and eliminates observer bias, yielding continuous, high-quality data that enable a more accurate evaluation of body posture and its effects on musculoskeletal health^20,21^. Such quantitative posture assessment tools enable objective evaluation of postural load associated with diverse ergonomic and environmental conditions, thereby supporting a more comprehensive understanding of MSDs-related risk factors. Investigating students’ posture, particularly during study activities, is of particular importance, as students often spend prolonged periods in static positions, such as sitting at a desk, which can contribute to the development or exacerbation of musculoskeletal disorders^22^. A detailed examination of various ergonomic and environmental factors, such as illuminance level, and their impact on students’ posture during study activities, particularly through image-based analysis methods such as Digimizer software, can contribute to identifying and improving ergonomic conditions and reducing long-term risks among students^23^. Owing to its ability to generate accurate and quantitative data on body angles, Digimizer enables more detailed and well-documented analyses and facilitates a more precise evaluation of factors affecting individuals’ posture and musculoskeletal health^24^.

Studies investigating the influence of physical factors on students’ posture, particularly in Iran, have been limited, mainly due to the high cost of equipment and the need for specialized expertise to implement and analyze direct and instrument-based methods. Consequently, most available studies have relied on paper-based assessment approaches. In this context, the present study, using image-based analysis, examines neck and trunk posture among students and its association with multiple ergonomic and environmental factors in study halls. This approach enables the extraction of accurate quantitative data on body angles and allows for the assessment of the effects of lighting on posture without the need for complex and costly equipment.

Given the multifactorial nature of musculoskeletal disorders and the importance of objective posture assessment, further investigation is needed to clarify how various contributing factors are reflected in students’ neck and trunk posture during study activities. the present study aimed to evaluate factors contributing to musculoskeletal disorders through quantitative analysis of neck and trunk posture among students of Shiraz University of Medical Sciences using Digimizer software.

## Materials and methods

### Participants

The participants in this study were students of Shiraz University of Medical Sciences who voluntarily took part after receiving a full explanation of the study objectives. Eligible participants were students present in the study halls during data collection, engaged in study activities at the time of assessment, and who provided written informed consent, with no self-reported history of musculoskeletal disorders affecting the neck or trunk. All participants completed a written informed consent form prior to enrollment. Measurements were conducted on 25 participants in late June, during the spring season, and throughout the night. Sample selection was carried out using a cluster sampling method, in which study halls from different faculties were considered as clusters, and the number of participants selected from each hall was determined based on the proportion of students in the corresponding faculty. Subsequently, participants within each cluster were selected using simple random sampling. Demographic information, including age, sex, height, weight, marital status, smoking status, level of physical activity, academic term and degree level, daily study duration, and rest patterns during study, was collected through a short questionnaire.

### Assessment of general and task lighting and luminance

To assess general lighting, the study area was divided into a regular grid, and illuminance was measured at the center of each grid cell at a height of one meter above the work surface. Measurements were performed using a TES-1339 lux meter, and after verifying the proper functioning of the device, the recorded values were compared with the Iranian national lighting standards. Task lighting was determined by measuring illuminance at several predefined points on the desk surface and calculating their mean value. Work surface luminance was measured using a photometer after stabilization of lighting conditions, at a standard observer distance, and recorded in cd/m2. Lighting uniformity was calculated as the ratio of minimum to average illuminance (Emin/Eavg) across the measurement grid and was evaluated in accordance with EN 12464-1:2021 lighting standards^25^.

### Assessment of participants’ posture

To evaluate participants’ posture, a combination of image-based analysis and complementary ergonomic assessment tools was employed to obtain a detailed representation of sitting posture and body alignment during study activities. Posture recording and analysis were conducted according to a standardized protocol under uniform environmental conditions. Participants were assessed in their usual sitting posture during study activities. Photographs were taken from lateral and posterior views using a fixed camera positioned at a predefined and consistent distance and orientation for all participants, with the camera aligned perpendicular to the ground. Multiple images were captured in each view, and the image with the clearest visibility of anatomical landmarks was selected for analysis. Images were obtained in a manner that allowed clear visualization of anatomical landmarks, including the earlobe, the C7 vertebra, the shoulders, and the pelvis. Following image acquisition, the files were transferred to Digimizer software, and neck and trunk angles were measured using the angle analysis tools. The neck angle was calculated based on the line extending from the earlobe to the C7 vertebra relative to a vertical reference line, while the trunk angle was determined based on the deviation of the trunk from the vertical axis. All measurements were performed by a trained assessor, and each angle was measured more than once, with the average value used for analysis.

### Assessment of musculoskeletal disorders

To complement the evaluation of body posture, an ergonomics specialist completed a desk and chair ergonomics questionnaire for each participant. This questionnaire was developed based on ergonomic principles and expert judgment and consisted of 12 dichotomous (yes/no) items related to the dimensions and fit of the desk and chair, as well as observed postural patterns. The checklist included items such as appropriateness of seat height, seat depth and width, backrest support, foot placement and support, suitability of desk height, under-desk clearance, availability of an appropriate work surface slope, and the presence of neck rotation, back flexion, and lateral body deviation. The results of this checklist were used to evaluate ergonomic conditions for subsequent analysis.

The Cornell Musculoskeletal Discomfort Questionnaire (CMDQ) was used to assess musculoskeletal disorders. The CMDQ is a standardized and validated instrument widely used in ergonomics research and occupational health assessments to evaluate musculoskeletal discomfort in work-related settings. This questionnaire is designed to identify and quantify discomfort intensity, frequency of symptoms, and the extent to which discomfort affects daily activities and work tasks over a weekly period, using a body map divided into 12 body regions. The validity and reliability of the Persian version of this questionnaire were confirmed in Iran by Afifehzadeh Kashani et al. in 2011, with a Cronbach’s alpha of 0.986^26^. Scoring for each body region is calculated as the product of the three aforementioned dimensions, yielding a minimum score of 0, indicating no discomfort, and a maximum score of 90, indicating the highest level of discomfort and impact. The sum of scores across the 12 body regions provides an overall representation of an individual’s musculoskeletal status and enables more precise identification of critical areas.

### Assessment of mental and cognitive workload

To evaluate the level of mental and cognitive workload experienced by participants, a single item derived from the NASA Task Load Index (NASA-TLX) questionnaire was used as a measure of perceived mental demand^27^. The Persian version of the NASA-TLX has been previously validated in Iran, demonstrating acceptable validity and reliability (Cronbach’s α = 0.897)^28^. Specifically, the item “How much mental and cognitive activity is required to perform the study task?”, was applied on a scale ranging from very low (0) to very high (20) to assess participants’ mental workload during study activities.

### Statistical analysis

Data analysis was performed using SPSS version 18. Descriptive statistics, including mean, standard deviation, frequency, and percentage, were used to summarize demographic, lighting, and posture variables. The Kolmogorov–Smirnov test was applied to assess data normality. Based on data distribution, independent t-test and one-way ANOVA or Mann–Whitney U and Kruskal–Wallis tests were employed as appropriate. Associations between lighting parameters and neck and trunk posture angles were examined using Pearson or Spearman correlation coefficients. Multiple linear regression analysis was conducted to evaluate the relationships between lighting characteristics and posture angles. A significance level of *p* < 0.05 was considered statistically significant.

## Results

**Fig. 1:**
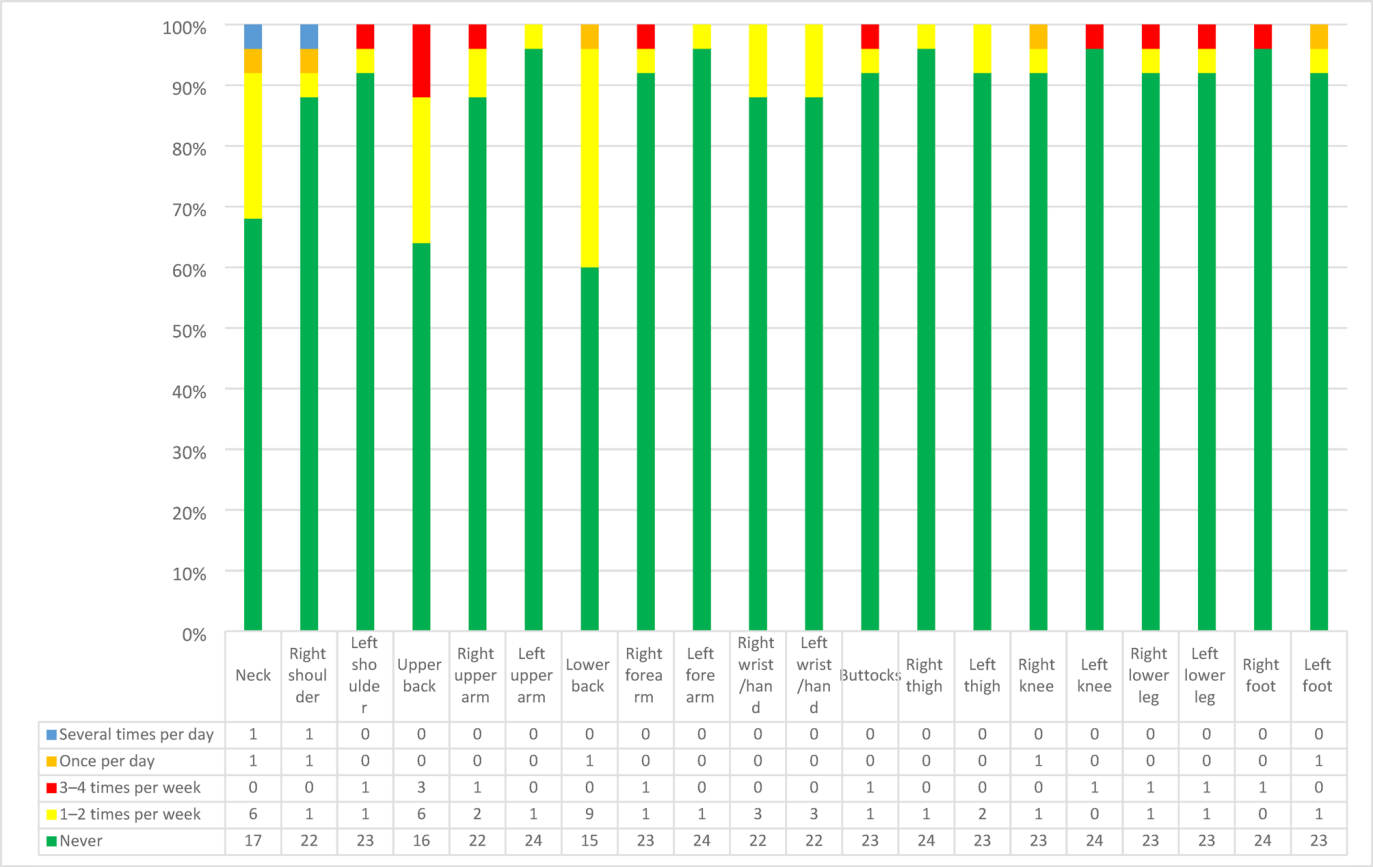
Distribution of pain frequency across different body regions based on Cornell questionnaire data.

**Fig. 2:**
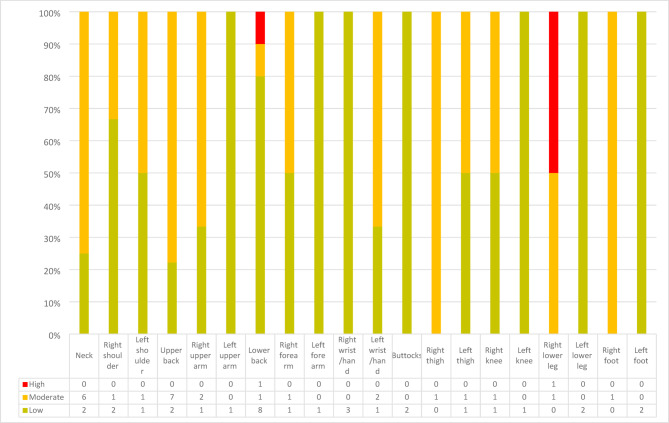
Distribution of pain duration across different body regions based on Cornell questionnaire data.

**Fig. 3:**
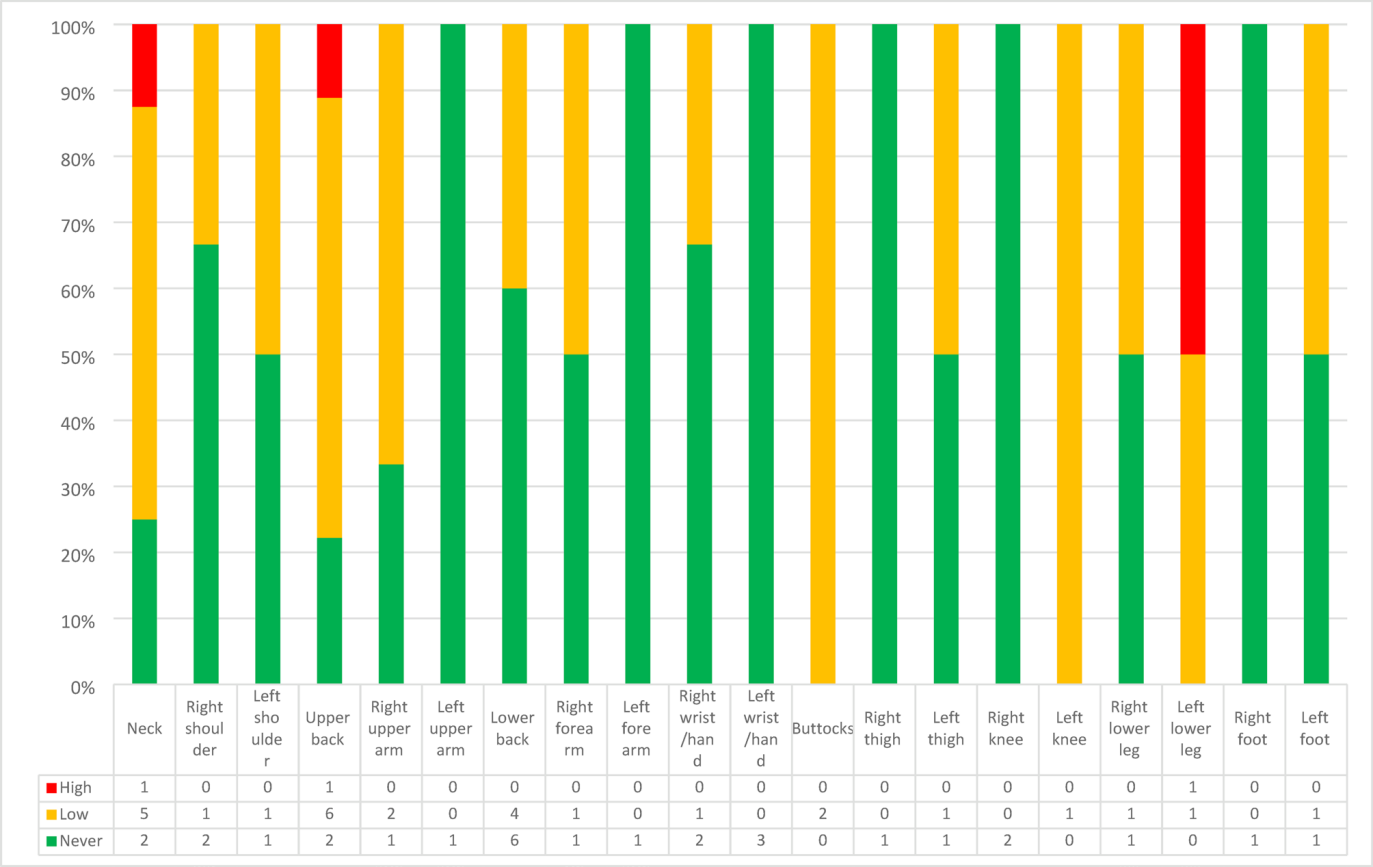
Distribution of the impact of pain and discomfort on students’ work ability based on Cornell questionnaire data.

This descriptive–analytical cross-sectional study was conducted among 25 students of Shiraz University of Medical Sciences. The findings indicated considerable variability in individual characteristics and study environment conditions among participants. The mean age of the students was 23.2 ± 4.28 years, ranging from 19 to 27 years. In terms of sex distribution, 80% of participants were male and 20% were female. Regarding marital status, 96% were single and only 4% were married. The mean height and weight of the participants were 173.72 ± 7.87 cm and 69.32 ± 9.36 kg, respectively, indicating a normal body mass index (BMI) with a mean value of 22.89 ± 2.07. In terms of lifestyle characteristics, 88% of the students did not smoke, while 12% reported tobacco use. In addition, 32% engaged in regular physical activity, and 84% reported taking periodic breaks during study. The mean daily study duration was 3.64 h, ranging from 1 to 7.5 h. The detailed results are presented in Table 1. The mean general lighting level in the study halls was 400.55 ± 12.6 lux, while the mean task lighting level was 409.74 ± 82.57 lux. The mean luminance of the desk surfaces was 31.03 cd/m^2^, and the lighting uniformity index was measured as 0.54. These values indicate that lighting conditions across the study halls and desks were satisfactory in terms of both illuminance and uniformity. Furthermore, analysis of pain frequency based on the Cornell questionnaire indicated that students reported the highest levels of pain intensity, duration of discomfort, and impact on work ability in the neck, upper back, and lower back regions, reflecting a considerable prevalence of musculoskeletal discomfort in the axial and upper body areas among students. In terms of pain frequency, discomfort was most frequently reported in the lower back (10 students), upper back (9 students), and neck (8 students). The upper back and neck regions were also associated with a moderate duration of discomfort (7 and 6 students, respectively) and a mild impact on students’ work ability (6 and 5 students, respectively). The results of these analyses are illustrated in Figs. 1, 2, 3.

**Table 1:** Correlation between participants’ demographic characteristics and neck and trunk angles (n = 25).

Variable	Mean ± SD	Minimum	Maximum	Neck		Trunk	
				r	*p*-Value	r	*p*-Value
Age (years)	23.2 ± 4.28	20	35	0.051	0.807	− 0.109	0.603
Height (cm)	173.72 ± 7.87	154	185	0.243	0.241	0.291	0.158
Weight (kg)	69.32 ± 9.36	45	88	0.255	0.218	0.226	0.278
BMI (kg/m^2^)	22.89 ± 2.07	18.73	27.77	0.151	0.471	0.082	0.697
Task lighting (lux)	409.74 ± 82.57	138.67	503.33	0.150	0.474	0.315	0.125
Luminance (cd/m^2^)	31.03 ± 5.65	18.73	39.97	− 0.060	0.776	− 0.006	0.979
General lighting (lux)	400.55 ± 12.6	170	662	− 0.217	0.298	− 0.363	0.074
Lighting uniformity	0.54 ± 0.1	0.42	0.64	0.080	0.705	0.246	0.235
NASA-TLX	12.4 ± 4.14	5	20	0.063	0.765	− 0.088	0.677
Daily study (hours/day)	3.64 ± 1.92	1	7.50	− 0.197	0.344	0.130	0.535

Variable		No. (%)	Neck		Trunk	
			Mean ± SD	*p*-value	Mean ± SD	p-value
Gender	Male	20(80)	39.28 ± 17.28	.141	12.92 ± 6.62	.020
	Female	5(20)	26.31 ± 15.76		7.51 ± 3.21	
Marital status	Single	24(96)	36.5 ± 17.84	.226	11.97 ± 6.53	< 0.001**
	Married	1(4)	41.03 ± 0		8.73 ± 0	
Smoking	Yes	3(12)	45.71 ± 4.91	.352	8.72 ± 1.48	.050
	No	22(88)	35.45 ± 18.28		12.26 ± 6.74	
Academic level	Bachelor’s degree	3(12)	36.92 ± 20.25	.930	12.05 ± 7.82	.794
	Master’s degree	0(0)	–		–	
	Doctoral degree	22(88)	36.26 ± 12.18		11.46 ± 3.01	
Study breaks	Yes	21(84)	35.64 ± 18.62	.507	12.54 ± 6.78	.016
	No	4(16)	42.15 ± 9.32		8.15 ± 1.59	
Daily physical activity	Yes	17(78)	32.82 ± 21.18	.461	11.97 ± 7.95	.946
	No	8(32)	38.5 ± 15.86		11.77 ± 5.85	

*BMI* Body Mass Index; *p*-values were obtained using Pearson and Spearman correlation analyses, Independent Samples t-test, and Mann–Whitney U test.

The findings of this study showed that none of the demographic variables were significantly correlated with neck angle (*p* > 0.05). Among demographic factors, only sex, marital status, and rest during study showed significant associations with trunk angle (*p* < 0.05), such that female and married participants exhibited smaller trunk angles during study. These results are presented in Table 1. The study also demonstrated that general lighting, task lighting, luminance, and the lighting uniformity index were not significantly correlated with trunk angles (*p* > 0.05). Further analysis of the associations between chair assessment items and neck and trunk angles showed that, among the five chair-related questions, inappropriate seat height was associated with an increase in neck angle (*p* = 0.046). In addition, inadequate seat width (*p* = 0.009) and lack of lower limb support or absence of a footrest were associated with an increase in trunk angle (*p* = 0.020). The results related to desk assessment items indicated that none of the variables examined in the questionnaire were associated with increased neck or trunk angles (*p* > 0.05). Moreover, the findings of posture-related items showed that participants with neck rotation (20% of participants) and lateral bending (16% of participants) did not exhibit significant changes in neck or trunk angles (*p* > 0.05). However, participants with trunk flexion (36% of participants) demonstrated a significant increase in trunk angle (*p* = 0.035). The results of these analyses are presented in Table 2. To examine the simultaneous effects of demographic variables, lighting indices, and mental workload on neck and trunk angles, multiple linear regression analysis was performed in two stages. Table 3 presents the analysis of factors associated with neck angle, in which the concurrent effects of quantitative variables including age, height, weight, daily study duration, academic term, NASA-TLX score, and various lighting indices were evaluated. In this study, to assess the assumption of no multicollinearity among independent variables, the Variance Inflation Factor (VIF) was calculated for each variable. The results indicate that the VIF values for all predictor variables fall below the threshold of 5, suggesting the absence of significant multicollinearity issues in the regression model. The results indicated that none of the variables included in this regression model had a significant linear effect on predicting changes in neck angle (*p* > 0.05). In Table 4, the effects of demographic variables, lighting indices, and mental workload on trunk angle were examined. The findings showed that none of the variables under investigation had a significant linear effect on changes in trunk angle (*p* > 0.05). Therefore, demographic characteristics, lighting indices, and NASA-TLX mental workload were not significant predictors of neck or trunk angles.

**Table 2:** Association between ergonomics assessment items and neck and trunk angles.

Questions			N (%)	Neck		Trunk	
				Mean ± SD	*p*-Value	Mean ± SD	*p*-Value
Chair	Is the seat height appropriate for the user, allowing a knee angle of approximately 90°?	Yes	19(76%)	32.8 ± 14.94	.046	12.64 ± 6.96	.273
		No	6(24%)	48.98 ± 20.62		9.28 ± 3.69	
	Is the seat depth appropriate for the user, with sufficient clearance between the seat edge and the popliteal region?	Yes	18(72%)	33.08 ± 15.33	.099	12.51 ± 7.14	.410
		No	7(28%)	45.95 ± 20.46		10.09 ± 4	
	Is the seat width appropriate for the user, providing adequate lateral clearance relative to hip width?	Yes	24(96%)	36.17 ± 17.67	.486	11.18 ± 5.65	.009
		No	1(4%)	48.95 ± 0		27.57 ± 0	
	Is the chair backrest appropriate, providing adequate support for the spine, particularly the lumbar region?	Yes	16(64%)	33.42 ± 14.79	.221	10.95 ± 5.79	.372
		No	9(36%)	42.48 ± 21.17		13.4 ± 7.54	
	During chair use, are the feet adequately supported on the floor or by a footrest?	Yes	5(20%)	26.31 ± 15.76	.141	7.51 ± 3.21	.020
		No	20(80%)	39.28 ± 17.28		12.92 ± 6.62	
Desk	Is the study desk height appropriate for the user, aligned with elbow height in the seated position?	Yes	19(76%)	39.88 ± 17.54	.105	12.81 ± 6.79	.184
		No	6(24%)	26.55 ± 14.11		8.76 ± 4.19	
	Does the desk surface provide sufficient workspace for the user?	Yes	22(88%)	37.08 ± 17.92	.765	12.22 ± 6.72	.425
		No	3(12%)	33.76 ± 16.89		8.99 ± 2.76	
	Is the desk surface adjustable in tilt to accommodate the user’s stature?	Yes	0(0%)	-	NS	–	NS
		No	25(100%)	36.68 ± 17.49		11.84 ± 6.43	
	Is there sufficient space under the desk for lower limb movement?	Yes	21(84%)	35.86 ± 19.02	.245	11.91 ± 6.85	.895
		No	4(16%)	40.99 ± 2.17		11.44 ± 4.12	
Posture	Does the individual exhibit neck rotation?	Yes	5(20%)	36.33 ± 15.58	.961	9.62 ± 5.05	.400
		No	20(80%)	36.77 ± 18.31		12.39 ± 6.72	
	Does the individual exhibit trunk flexion?	Yes	9(36%)	28.72 ± 18.11	.088	15.38 ± 7.75	.035
		No	16(64%)	41.16 ± 15.97		9.84 ± 4.72	
	Does the individual exhibit lateral trunk flexion?	Yes	4(16%)	31.56 ± 13.1	.534	9.22 ± 5.74	.385
		No	21(84%)	37.66 ± 18.3		12.33 ± 6.56	

*NS* Non significant.

**Table 3:** Multiple linear regression analysis of neck angle based on the studied variables.

Variables	β	SE	t	*p*-value	95% CI	
					Lower	Upper
Age	− 0.380	2.058	− 0.754	0.466	− 5.966	2.863
Height	− 0.099	1.708	− 0.128	0.904	− 3.883	3.444
Weight	0.230	1.190	0.362	0.713	− 2.121	2.982
Daily study duration (hours)	− 0.461	4.491	− 0.935	0.365	− 13.838	5.427
Academic term	− 0.243	2.519	− 0.744	0.469	− 7.279	3.525
NASA-TLX	0.238	1.522	0.660	0.524	− 2.260	4.269
General lighting (lux)	− 0.948	2.125	− 0.619	0.541	− 5.874	3.243
Task lighting (lux)	− 0.011	0.085	− 0.028	0.972	− 0.185	0.180
luminance (cd/m^2^)	− 0.272	0.942	− 0.892	0.387	− 2.864	1.180
Lighting uniformity	− 0.990	243.632	− 0.706	0.492	− 694.576	350.500
Study breaks	0.967	0.711	0.540	0.242	− 0.211	2.718

**Table 4:** Multiple linear regression analysis of trunk angle based on the studied variables.

Variables	β	SE	t	*p*-value	95% CI	
					Lower	Upper
Age	0.067	0.739	0.136	0.893	− 1.484	1.685
Height	− 0.583	0.613	− 0.776	0.450	− 1.791	0.839
Weight	0.184	0.427	0.296	0.772	− 0.789	1.042
Daily study duration (hours)	0.628	1.612	1.301	0.214	− 1.360	5.555
Academic term	− 0.006	0.904	− 0.018	0.986	− 1.956	1.923
NASA-TLX	0.088	0.546	0.250	0.806	− 1.035	1.308
General lighting (lux)	− 1.027	0.763	− 0.686	0.504	− 2.160	1.113
Task lighting (lux)	0.097	0.031	0.246	0.809	− 0.058	0.073
luminance (cd/m^2^)	0.052	0.338	0.175	0.864	− 0.666	0.784
Lighting uniformity	− 0.116	87.446	− 0.085	0.934	− 194.954	180.153

## Discussion

This study aimed to investigate factors influencing neck and trunk posture among students during study activities, with a particular focus on ergonomic characteristics, individual factors, and selected environmental conditions. By integrating quantitative posture assessment using image-based analysis with measurements of lighting indices and ergonomic features of desks and chairs, the study enabled a multidimensional evaluation of posture-related risk factors. The application of Digimizer software allowed for precise and objective quantification of neck and trunk angles, providing a robust basis for interpreting the relative contribution of different factors to observed posture patterns. The findings of the present study showed that demographic variables, including age, height, weight, daily study duration, and academic term, did not contribute to variations in neck angle, and that neck and trunk angles were determined independently of individual characteristics. However, the potential influence of variables not retained in the final regression model, such as physical activity, cannot be entirely ruled out and may have contributed to residual confounding.

These results are consistent with the findings reported by Fraulin et al., who indicated that, except for body weight, other variables such as age, sex, and BMI do not have a significant effect on biomechanical indices^29^. Similarly, studies by Guduru and Mohipour demonstrated that observed angular differences between groups persisted even after controlling for individual characteristics, indicating that the primary source of posture variation is not demographic factors but biomechanical mechanisms and body alignment strategies^30,31^. The findings of Nourollahi-Darabad further support these results, showing that the influence of anthropometric differences becomes prominent only when chair dimensions are incompatible with users’ body characteristics^32^.

General and task lighting indices, luminance, and lighting uniformity were all within standard ranges, and none of these variables showed a significant association with neck or trunk angles. Regression analysis further indicated that none of the lighting components were able to predict angular changes in body posture, and that postural behavior under adequate lighting conditions was primarily influenced by chair design and body alignment rather than lighting characteristics. The findings of Merbah demonstrated that under standard lighting conditions, neck posture is mainly affected by mechanical factors, and lighting-related angular changes are not observed^33^. Similarly, Yan reported that postural stability is influenced by lighting only when illuminance level or correlated color temperature deviates from optimal ranges, whereas under appropriate lighting conditions, body posture remains stable^34^. In contrast, evidence from studies by Silva and Dianat showed that exposure to lighting levels below recommended standards leads to increased neck flexion and angular changes in the trunk, with participants adopting compensatory strategies to improve visual performance^35,36^. Findings from studies by Zamanian and Pirmoradi also indicated that substandard lighting is associated with increased neck angle and higher ergonomic risk scores^11,17^.

The discrepancy between these studies and the present findings can be attributed to differences in lighting conditions. In the current study, lighting indices were within recommended limits; therefore, compensatory mechanisms related to insufficient lighting were not activated. Karami similarly reported that lighting did not play a decisive role in postural changes, and that mismatch between desk and chair dimensions and users’ anthropometric characteristics was the primary contributor to postural deviations^20^. The results of the Cornell questionnaire indicated that the highest levels of pain, duration of discomfort, and impact on work ability were reported in the neck, upper back, and lower back regions. In addition, the upper back and neck exhibited the greatest duration of discomfort at a moderate level, and these two regions had the strongest impact on students’ work ability. This pattern suggests that musculoskeletal discomfort is most prevalent in the axial regions of the body, with postural loads resulting from prolonged sitting predominantly accumulating in the neck and trunk. These findings are consistent with studies by Mohipour and Nourollahi-Darabad, which reported that the trunk and neck are the regions with the highest frequency of discomfort and that axial body segments show the greatest sensitivity to postural deviations^30,32^. The concordance of these findings indicates that during seated activities, cumulative loads arising from trunk flexion and the maintenance of static postures lead to the greatest discomfort in the neck and trunk, making these regions the first to exhibit clinically meaningful responses when postural stability is compromised.

The findings of the present study showed that inappropriate seat height was associated with an increase in neck angle, while insufficient seat width, lack of lower limb support, and absence of a footrest were associated with an increase in trunk angle. These findings indicate that chair dimensions and the level of ergonomic support play a decisive role in shaping posture patterns. Existing evidence also confirms that mismatch between chair dimensions and users’ anthropometric characteristics is associated with increased neck and trunk angles and imposes greater mechanical load on the axial regions of the body^11,17,33–35,37,38^. It was also observed that neck rotation and lateral bending were not associated with changes in body angles, whereas the presence of trunk flexion was associated with an increased trunk angle. Previous studies have similarly reported that postural changes in the sagittal plane, particularly forward flexion, are the primary contributors to increased spinal loading, and that lateral deviations become influential only when accompanied by trunk or neck flexion^30,32,37,39^. The consistency of these findings indicates that during seated activities, cumulative loads are predominantly driven by trunk flexion, while the role of lateral movements is limited.

The present study further demonstrated that insufficient support in either the upper or lower extremities compels the body to adopt greater angular adjustments in the neck and trunk to maintain postural stability. Previous research has likewise shown that lack of support surfaces, improper adjustment of seat height or width, and inadequate lower limb space lead to flexed postures and increased muscular activity in the neck and trunk regions^29,30,32,38,40^. This mechanism is consistent with the findings of the present study. The NASA-TLX mental workload variable did not show a linear effect on predicting changes in neck and trunk angles in the regression analysis. Yan and colleagues reported that lighting quality and visual conditions can act as stimuli for increased mental effort, and that changes in illuminance level or correlated color temperature may alter the level of attention and cognitive load required to perform tasks^34^. The discrepancy between the findings of the present study and those reported by Yan may be attributed to the fact that, in the current study, lighting indices were within standard ranges and visual conditions did not impose additional strain. Consequently, mechanisms leading to increased mental workload were not activated, and posture patterns developed independently of cognitive demands.

In the present study, Digimizer software was used to extract neck and trunk angles. This approach enabled precise identification of anatomical landmarks and allowed for quantitative and repeatable recording of angles under real study conditions. The image-based nature of this method ensured that angle measurements were independent of observer judgment and that even subtle changes in head and trunk posture could be detected. Because postural outcomes in this study were derived from these accurate measurements, the use of Digimizer played a critical role in ensuring measurement validity and enabled detailed analysis of the effects of chair design on posture patterns. Previous evidence has also demonstrated that image-based analysis tools provide higher accuracy than observational methods and are capable of capturing small angular changes, whereas observational tools such as RULA or ROSA do not adequately reflect subtle deviations in neck and trunk posture^11,17^. In addition, studies using instrumented tools such as inclinometers or goniometers have reported that although these devices offer high accuracy in controlled environments, they often limit the ability to capture natural body posture in real-world settings^30,32^. In the present study, Digimizer occupied an intermediate position between these two categories of tools by combining the measurement precision of instrumented methods with the ability to record posture under natural study conditions, thereby allowing assessment of angle deviations related to chair design without environmental interference. Furthermore, Digimizer-based studies have reported that this tool is capable of accurately extracting angles related to head, shoulder, and trunk posture and is well suited for field studies in which both measurement accuracy and ecological validity are essential^31^.

## Conclusion

The results of this study demonstrated that students’ posture patterns during seated activities are more strongly determined by ergonomic factors related to the chair and study workstation than by individual characteristics or mental workload. Increases in neck angle occurred primarily when seat height was incompatible with users’ anthropometric requirements, while increases in trunk angle were associated with insufficient lower limb support and inappropriate seat dimensions. Lighting conditions were within standard ranges and did not contribute to angular changes in body posture, and mental workload was not included as a significant predictor in the regression models.

These findings indicate that posture deviations during seated activities, even in the presence of individual differences or varying levels of cognitive demand, arise mainly when chair components and workspace design are not aligned with users’ body characteristics. The use of Digimizer-based image analysis enabled precise identification of these deviations and demonstrated that even small mismatches in chair dimensions and support can lead to increased neck and trunk flexion angles. Overall, these findings underscore the importance of designing educational furniture that accommodates the anthropometric diversity of students and of applying ergonomic principles in study environments.

### Limitations

This study has several limitations that should be considered when interpreting the findings. The relatively small sample size may have limited the statistical power to detect subtle associations. Moreover, the study was conducted under typical educational conditions with lighting parameters within recommended standards, and thus the findings primarily reflect posture behavior in adequately lit study environments. In addition, mental workload was assessed using a single-item measure derived from the NASA-TLX, which may not fully capture the multidimensional nature of mental workload.

## Data Availability

The datasets used and analysed during the current study available from the corresponding author on reasonable request.
